# (1*SR*,2*RS*,3*SR*,5*SR*,6*RS*)-6-[(*Z*)-1-Acet­oxy-2-phenyl­ethen­yl]-3-eth­oxy-2-phenyl­bicyclo­[3.1.0]hexan-1-yl acetate

**DOI:** 10.1107/S1600536811001760

**Published:** 2011-01-29

**Authors:** Wen-Xiang Hu, Gao Xu, Guo-Qiang Wei

**Affiliations:** a Department of Chemistry, Capital Normal University, Beijing 100048, People’s Republic of China

## Abstract

The mol­ecule of the title compound, C_26_H_28_O_5_, is chiral with five stereogenic centres; however, the centrosymmetric triclinic group gives a racemic crystal. The fused ring system adopta boat conformation in which the cyclo­propane ring plane is roughly perpendicular to the styryl group plane, forming a dihedral angle of 74.78 (19)°. The dihedral angle between the two benzene rings is 77.24 (6)°.

## Related literature

For related structures, see: Li *et al.* (2008 ▶); Zhang *et al.* (2008 ▶). For general backgound to the bicyclo­[3.1.0]hexane unit, see: Donaldson (2001 ▶); Ezzitouni & Marquez (1997 ▶); Hanessian *et al.* (1995 ▶); Monn *et al.* (1997 ▶).
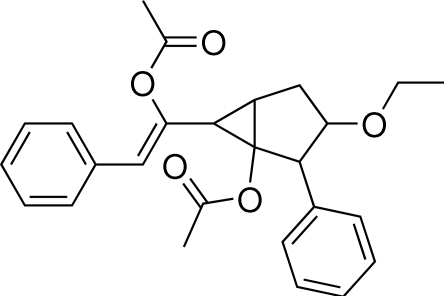


## Experimental

### 

#### Crystal data

C_26_H_28_O_5_
*M* *_r_* = 420.48
Triclinic, 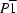

*a* = 5.8585 (14) Å
*b* = 12.368 (3) Å
*c* = 15.852 (4) Å
α = 73.170 (6)°
β = 88.967 (9)°
γ = 81.761 (7)°
*V* = 1087.7 (4) Å^3^
*Z* = 2
Mo *K*α radiation
μ = 0.09 mm^−1^
*T* = 103 K
0.43 × 0.37 × 0.10 mm

#### Data collection

Rigaku AFC10/Saturn724+ diffractometer
10211 measured reflections
4888 independent reflections
2841 reflections with *I* > 2σ(*I*)
*R*_int_ = 0.053

#### Refinement

*R*[*F* ^2^ > 2σ(*F* ^2^)] = 0.048
*wR*(*F* ^2^) = 0.076
*S* = 0.94
4888 reflections
277 parameters
H-atom parameters constrained
Δρ_max_ = 0.58 e Å^−3^
Δρ_min_ = −0.32 e Å^−3^

### 

Data collection: *CrystalClear* (Rigaku, 2008) ▶; cell refinement: *CrystalClear* ▶; data reduction: *CrystalClear* ▶; program(s) used to solve structure: *SHELXS97* (Sheldrick, 2008 ▶); program(s) used to refine structure: *SHELXL97* (Sheldrick, 2008 ▶); molecular graphics: *DIAMOND* (Brandenburg, 1999 ▶); software used to prepare material for publication: *publCIF* (Westrip, 2010 ▶).

## Supplementary Material

Crystal structure: contains datablocks global, I. DOI: 10.1107/S1600536811001760/kp2298sup1.cif

Structure factors: contains datablocks I. DOI: 10.1107/S1600536811001760/kp2298Isup2.hkl

Additional supplementary materials:  crystallographic information; 3D view; checkCIF report

## Figures and Tables

**Fig. 1.**
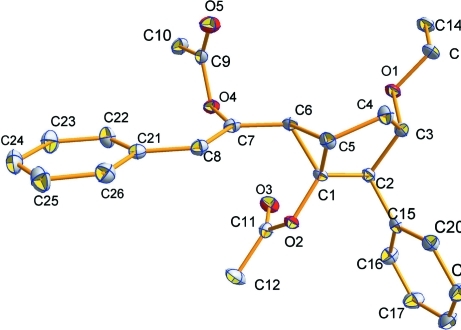
Molecular structure of the title compound showing atom labeling scheme and displacement ellipsoids at the 50% probability level. Hydrogen atoms are omitted for clarity.

**Fig. 2.**
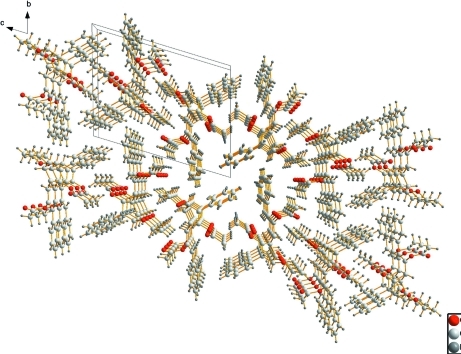
The three-dimensional structure of the title compound. The crystal packing is defined by van der Waals interactions.

## supplementary crystallographic information

### Comment

Small-ring compounds and their derivatives arouse considerable interest because their energy content relative to their acyclic counterparts often results in unexpected properties. Bicyclo[3.1.0]hexane, which contained a cyclopropane unit, is an important structural element in a wide range of naturally occurring compounds (Hanessian *et al.*, 1995; Donaldson, 2001), and as a privileged unit in medicinal chemistry (Monn *et al.*, 1997; Ezzitouni *et al.*, 1997) since it possesses unique stereochemical and electronic properties in conjunction with high metabolic stability. In this paper, we will report the structure of the title compound, a new polysubstituted bicyclo[3.1.0]hexane compound, which was simply prepared from the gold catalysed reaction of vinyl ether with propargylic ester.

The title molecule (Fig.1), is mainly composed of two fused rings A (C1—C5—C6), and B (C1—C2—C3—C4—C5). The ring B has an envelope conformation, C3 and ring A lie to the same side of the plane defined by C1—C2—C4—C5. The ring A is roughly perpendicular to the styryl group and almost parallels the cyclopentane-attached benzene ring. The dihedral angle between ring A and the styryl group is 74.782 (99)°, while the dihedral angle data between two benzene rings is 77.235 (58)°.

### Experimental

Under an atmosphere of nitrogen, IprAuNTf_2_(12.92 mg, 0.0141 mmol) was added to a solution of 3-phenyl-1-propyn-3-yl acetate (98.46 mg, 0.565 mmol) and vinyl ethyl ether (0.8 ml) in 11 ml dry 1,2-dichloroethane. After stirring for 3 h, the solution was concentrated *in vacuo*. The crude product was purified by flash chromatography on silica gel (diethyl ether:n-hexane = 1:20) to give the title compound as a white solid, which was then recrystallized from EtOAc and pentane (EtOAc:pentane = 1:9) to afford a colourless platee-like crystals.

### Refinement

Carbon-bound H-atoms were placed in calculated positions(C—H=0.95 to 1.00 Å), respectively, and constrained to ride on their parent atoms with *U*_iso_(H) set to 1.2–1.5 U_equiv_(C).

### Crystal data

**Table:**

C_26_H_28_O_5_	*Z* = 2
*M**_r_* = 420.48	*F*(000) = 448
Triclinic, *P*1	*D*_x_ = 1.284 Mg m^−^^3^
Hall symbol: -P 1	Mo *K*α radiation, λ = 0.71073 Å
*a* = 5.8585 (14) Å	Cell parameters from 3076 reflections
*b* = 12.368 (3) Å	θ = 3.3–27.5°
*c* = 15.852 (4) Å	µ = 0.09 mm^−^^1^
α = 73.170 (6)°	*T* = 103 K
β = 88.967 (9)°	Platelet, colourless
γ = 81.761 (7)°	0.43 × 0.37 × 0.10 mm
*V* = 1087.7 (4) Å^3^	

### Data collection

**Table:**

Rigaku AFC10/Saturn724+ diffractometer	2841 reflections with *I* > 2σ(*I*)
Radiation source: Rotating Anode	*R*_int_ = 0.053
graphite	θ_max_ = 27.5°, θ_min_ = 3.4°
Detector resolution: 28.5714 pixels mm^-1^	*h* = −7→7
φ and ω scans	*k* = −15→16
10211 measured reflections	*l* = −20→20
4888 independent reflections	

### Refinement

**Table:**

Refinement on *F*^2^	Primary atom site location: structure-invariant direct methods
Least-squares matrix: full	Secondary atom site location: difference Fourier map
*R*[*F*^2^ > 2σ(*F*^2^)] = 0.048	Hydrogen site location: inferred from neighbouring sites
*wR*(*F*^2^) = 0.076	H-atom parameters constrained
*S* = 0.94	*w* = 1/[σ^2^(*F*_o_^2^) + (0.0012*P*)^2^] where *P* = (*F*_o_^2^ + 2*F*_c_^2^)/3
4888 reflections	(Δ/σ)_max_ < 0.001
277 parameters	Δρ_max_ = 0.58 e Å^−^^3^
0 restraints	Δρ_min_ = −0.32 e Å^−^^3^

### Special details

**Table:**

Geometry. All e.s.d.'s (except the e.s.d. in the dihedral angle between two l.s. planes) are estimated using the full covariance matrix. The cell e.s.d.'s are taken into account individually in the estimation of e.s.d.'s in distances, angles and torsion angles; correlations between e.s.d.'s in cell parameters are only used when they are defined by crystal symmetry. An approximate (isotropic) treatment of cell e.s.d.'s is used for estimating e.s.d.'s involving l.s. planes.
Refinement. Refinement of *F*^2^ against ALL reflections. The weighted *R*-factor *wR* and goodness of fit *S* are based on *F*^2^, conventional *R*-factors *R* are based on *F*, with *F* set to zero for negative *F*^2^. The threshold expression of *F*^2^ > σ(*F*^2^) is used only for calculating *R*-factors(gt) *etc*. and is not relevant to the choice of reflections for refinement. *R*-factors based on *F*^2^ are statistically about twice as large as those based on *F*, and *R*- factors based on ALL data will be even larger.

### Fractional atomic coordinates and isotropic or equivalent isotropic displacement parameters (Å^2^)

**Table:**

	*x*	*y*	*z*	*U*_iso_*/*U*_eq_	
O1	0.21125 (19)	0.60902 (10)	0.37934 (8)	0.0188 (3)	
O2	0.45865 (19)	0.47059 (9)	0.15005 (8)	0.0165 (3)	
O3	0.1062 (2)	0.41550 (10)	0.16972 (9)	0.0256 (3)	
O4	0.34585 (18)	0.21945 (9)	0.34813 (8)	0.0171 (3)	
O5	0.5217 (2)	0.17190 (10)	0.48169 (8)	0.0232 (3)	
C20	0.4622 (3)	0.76441 (14)	0.12130 (12)	0.0190 (4)	
H20	0.5858	0.7499	0.1629	0.023*	
C19	0.4674 (3)	0.84861 (14)	0.04176 (12)	0.0234 (5)	
H19	0.5936	0.8907	0.0291	0.028*	
C18	0.2871 (3)	0.87087 (15)	−0.01919 (13)	0.0273 (5)	
H18	0.2879	0.9290	−0.0736	0.033*	
C17	0.1075 (3)	0.80827 (15)	−0.00027 (13)	0.0281 (5)	
H17	−0.0155	0.8225	−0.0421	0.034*	
C16	0.1049 (3)	0.72458 (14)	0.07945 (12)	0.0227 (5)	
H16	−0.0210	0.6823	0.0918	0.027*	
C15	0.2822 (3)	0.70114 (14)	0.14168 (12)	0.0167 (4)	
C2	0.2748 (3)	0.60988 (13)	0.22886 (11)	0.0157 (4)	
H2	0.1132	0.5928	0.2392	0.019*	
C3	0.3613 (3)	0.64292 (14)	0.30821 (12)	0.0172 (4)	
H3	0.3576	0.7276	0.2917	0.021*	
C4	0.6121 (3)	0.58431 (14)	0.32826 (12)	0.0187 (4)	
H4B	0.7208	0.6388	0.3026	0.022*	
H4A	0.6422	0.5548	0.3927	0.022*	
C5	0.6408 (3)	0.48698 (14)	0.28685 (11)	0.0162 (4)	
H5	0.7961	0.4607	0.2668	0.019*	
C1	0.4341 (3)	0.50053 (14)	0.23016 (12)	0.0151 (4)	
C6	0.4684 (3)	0.40331 (13)	0.31560 (11)	0.0159 (4)	
H6	0.3671	0.4142	0.3648	0.019*	
C7	0.5228 (3)	0.28429 (14)	0.31184 (12)	0.0165 (4)	
C8	0.7029 (3)	0.23893 (14)	0.27441 (11)	0.0170 (4)	
H8	0.8082	0.2900	0.2483	0.020*	
C21	0.7645 (3)	0.12233 (14)	0.26717 (11)	0.0170 (4)	
C22	0.6235 (3)	0.03660 (14)	0.29030 (12)	0.0223 (5)	
H22	0.4741	0.0525	0.3120	0.027*	
C23	0.6990 (3)	−0.07128 (15)	0.28197 (13)	0.0267 (5)	
H23	0.6016	−0.1287	0.2987	0.032*	
C24	0.9144 (3)	−0.09582 (16)	0.24957 (13)	0.0270 (5)	
H24	0.9652	−0.1698	0.2438	0.032*	
C25	1.0556 (3)	−0.01220 (15)	0.22559 (13)	0.0260 (5)	
H25	1.2032	−0.0282	0.2025	0.031*	
C26	0.9818 (3)	0.09516 (15)	0.23522 (12)	0.0213 (4)	
H26	1.0818	0.1515	0.2197	0.026*	
C11	0.2788 (3)	0.42436 (14)	0.12820 (13)	0.0197 (4)	
C12	0.3253 (3)	0.38905 (16)	0.04671 (13)	0.0330 (5)	
H12A	0.2224	0.3345	0.0432	0.050*	
H12B	0.4861	0.3531	0.0482	0.050*	
H12C	0.2980	0.4563	−0.0050	0.050*	
C13	0.2398 (3)	0.66215 (15)	0.44723 (12)	0.0233 (5)	
H13B	0.2472	0.7445	0.4205	0.028*	
H13A	0.3856	0.6268	0.4809	0.028*	
C14	0.0379 (3)	0.64685 (15)	0.50811 (12)	0.0252 (5)	
H14B	−0.1054	0.6839	0.4747	0.038*	
H14C	0.0576	0.6815	0.5553	0.038*	
H14A	0.0306	0.5652	0.5338	0.038*	
C9	0.3658 (3)	0.16383 (15)	0.43626 (13)	0.0232 (3)	
C10	0.1810 (3)	0.09224 (14)	0.46514 (12)	0.0238 (5)	
H10A	0.2242	0.0190	0.4527	0.036*	
H10B	0.0368	0.1314	0.4332	0.036*	
H10C	0.1596	0.0789	0.5286	0.036*	

### Atomic displacement parameters (Å^2^)

**Table:**

	*U*^11^	*U*^22^	*U*^33^	*U*^12^	*U*^13^	*U*^23^
O1	0.0232 (7)	0.0222 (7)	0.0138 (7)	−0.0053 (5)	0.0043 (6)	−0.0089 (5)
O2	0.0212 (7)	0.0178 (7)	0.0129 (7)	−0.0046 (5)	0.0043 (5)	−0.0074 (5)
O3	0.0225 (8)	0.0344 (8)	0.0250 (9)	−0.0116 (6)	0.0070 (6)	−0.0136 (6)
O4	0.0179 (7)	0.0180 (7)	0.0145 (7)	−0.0047 (5)	0.0036 (5)	−0.0027 (5)
O5	0.0286 (7)	0.0221 (6)	0.0192 (7)	−0.0072 (5)	0.0001 (5)	−0.0048 (5)
C20	0.0194 (11)	0.0189 (10)	0.0188 (11)	−0.0030 (8)	0.0000 (8)	−0.0055 (8)
C19	0.0256 (11)	0.0197 (10)	0.0247 (12)	−0.0077 (8)	0.0051 (9)	−0.0042 (8)
C18	0.0338 (12)	0.0198 (10)	0.0227 (12)	−0.0022 (9)	0.0013 (10)	0.0015 (8)
C17	0.0280 (12)	0.0317 (12)	0.0213 (13)	−0.0041 (10)	−0.0042 (9)	−0.0022 (9)
C16	0.0177 (10)	0.0239 (10)	0.0252 (12)	−0.0052 (8)	−0.0006 (9)	−0.0041 (9)
C15	0.0208 (10)	0.0139 (9)	0.0169 (11)	−0.0004 (8)	0.0028 (8)	−0.0079 (8)
C2	0.0177 (10)	0.0164 (9)	0.0154 (11)	−0.0055 (8)	0.0046 (8)	−0.0074 (8)
C3	0.0213 (11)	0.0168 (9)	0.0155 (11)	−0.0062 (8)	0.0031 (8)	−0.0062 (8)
C4	0.0177 (10)	0.0176 (10)	0.0215 (11)	−0.0019 (8)	0.0002 (8)	−0.0069 (8)
C5	0.0138 (10)	0.0177 (10)	0.0184 (11)	−0.0030 (8)	0.0032 (8)	−0.0073 (8)
C1	0.0195 (10)	0.0160 (9)	0.0113 (10)	−0.0027 (8)	0.0037 (8)	−0.0064 (7)
C6	0.0199 (10)	0.0165 (9)	0.0120 (10)	−0.0038 (8)	0.0056 (8)	−0.0048 (7)
C7	0.0172 (10)	0.0178 (10)	0.0135 (10)	−0.0050 (8)	0.0002 (8)	−0.0019 (8)
C8	0.0166 (10)	0.0173 (9)	0.0170 (11)	−0.0050 (8)	0.0017 (8)	−0.0035 (8)
C21	0.0186 (10)	0.0169 (10)	0.0147 (11)	−0.0017 (8)	−0.0016 (8)	−0.0040 (8)
C22	0.0187 (11)	0.0208 (10)	0.0288 (13)	−0.0017 (8)	0.0017 (9)	−0.0099 (9)
C23	0.0288 (12)	0.0196 (10)	0.0340 (13)	−0.0059 (9)	0.0003 (10)	−0.0104 (9)
C24	0.0286 (12)	0.0195 (10)	0.0351 (14)	0.0035 (9)	−0.0056 (10)	−0.0143 (9)
C25	0.0194 (11)	0.0274 (11)	0.0326 (13)	0.0034 (9)	−0.0017 (9)	−0.0139 (9)
C26	0.0209 (11)	0.0208 (10)	0.0223 (12)	−0.0031 (8)	0.0003 (9)	−0.0065 (8)
C11	0.0254 (12)	0.0168 (10)	0.0185 (11)	−0.0076 (8)	0.0024 (9)	−0.0056 (8)
C12	0.0468 (14)	0.0389 (13)	0.0250 (13)	−0.0217 (11)	0.0146 (10)	−0.0207 (10)
C13	0.0324 (12)	0.0245 (11)	0.0157 (11)	−0.0038 (9)	0.0010 (9)	−0.0104 (8)
C14	0.0306 (12)	0.0274 (11)	0.0188 (12)	0.0013 (9)	0.0018 (9)	−0.0114 (9)
C9	0.0286 (7)	0.0221 (6)	0.0192 (7)	−0.0072 (5)	0.0001 (5)	−0.0048 (5)
C10	0.0272 (11)	0.0192 (10)	0.0232 (12)	−0.0074 (8)	0.0055 (9)	−0.0014 (8)

### Geometric parameters (Å, °)

**Table:**

O1—C3	1.420 (2)	C1—C6	1.523 (2)
O1—C13	1.4372 (19)	C6—C7	1.479 (2)
O2—C11	1.363 (2)	C6—H6	1.0000
O2—C1	1.4215 (19)	C7—C8	1.334 (2)
O3—C11	1.198 (2)	C8—C21	1.473 (2)
O4—C9	1.366 (2)	C8—H8	0.9500
O4—C7	1.414 (2)	C21—C26	1.394 (2)
O5—C9	1.2057 (18)	C21—C22	1.398 (2)
C20—C15	1.381 (2)	C22—C23	1.387 (2)
C20—C19	1.387 (2)	C22—H22	0.9500
C20—H20	0.9500	C23—C24	1.381 (3)
C19—C18	1.387 (2)	C23—H23	0.9500
C19—H19	0.9500	C24—C25	1.380 (2)
C18—C17	1.373 (2)	C24—H24	0.9500
C18—H18	0.9500	C25—C26	1.387 (2)
C17—C16	1.384 (2)	C25—H25	0.9500
C17—H17	0.9500	C26—H26	0.9500
C16—C15	1.386 (2)	C11—C12	1.488 (2)
C16—H16	0.9500	C12—H12A	0.9800
C15—C2	1.515 (2)	C12—H12B	0.9800
C2—C1	1.524 (2)	C12—H12C	0.9800
C2—C3	1.545 (2)	C13—C14	1.511 (3)
C2—H2	1.0000	C13—H13B	0.9900
C3—C4	1.538 (2)	C13—H13A	0.9900
C3—H3	1.0000	C14—H14B	0.9800
C4—C5	1.518 (2)	C14—H14C	0.9800
C4—H4B	0.9900	C14—H14A	0.9800
C4—H4A	0.9900	C9—C10	1.479 (2)
C5—C1	1.482 (2)	C10—H10A	0.9800
C5—C6	1.519 (2)	C10—H10B	0.9800
C5—H5	1.0000	C10—H10C	0.9800
			
C3—O1—C13	112.11 (13)	C1—C6—H6	115.3
C11—O2—C1	114.87 (14)	C8—C7—O4	120.48 (15)
C9—O4—C7	116.41 (12)	C8—C7—C6	127.71 (17)
C15—C20—C19	121.78 (16)	O4—C7—C6	111.64 (15)
C15—C20—H20	119.1	C7—C8—C21	130.31 (17)
C19—C20—H20	119.1	C7—C8—H8	114.8
C20—C19—C18	119.51 (18)	C21—C8—H8	114.8
C20—C19—H19	120.2	C26—C21—C22	117.45 (16)
C18—C19—H19	120.2	C26—C21—C8	117.21 (16)
C17—C18—C19	119.49 (18)	C22—C21—C8	125.33 (17)
C17—C18—H18	120.3	C23—C22—C21	120.89 (18)
C19—C18—H18	120.3	C23—C22—H22	119.6
C18—C17—C16	120.24 (17)	C21—C22—H22	119.6
C18—C17—H17	119.9	C24—C23—C22	120.54 (18)
C16—C17—H17	119.9	C24—C23—H23	119.7
C17—C16—C15	121.40 (18)	C22—C23—H23	119.7
C17—C16—H16	119.3	C25—C24—C23	119.54 (17)
C15—C16—H16	119.3	C25—C24—H24	120.2
C20—C15—C16	117.56 (17)	C23—C24—H24	120.2
C20—C15—C2	121.91 (15)	C24—C25—C26	119.97 (19)
C16—C15—C2	120.53 (16)	C24—C25—H25	120.0
C15—C2—C1	111.54 (15)	C26—C25—H25	120.0
C15—C2—C3	113.55 (14)	C25—C26—C21	121.60 (17)
C1—C2—C3	103.93 (12)	C25—C26—H26	119.2
C15—C2—H2	109.2	C21—C26—H26	119.2
C1—C2—H2	109.2	O3—C11—O2	123.27 (17)
C3—C2—H2	109.2	O3—C11—C12	125.48 (17)
O1—C3—C4	113.68 (14)	O2—C11—C12	111.24 (16)
O1—C3—C2	108.36 (13)	C11—C12—H12A	109.5
C4—C3—C2	106.92 (14)	C11—C12—H12B	109.5
O1—C3—H3	109.3	H12A—C12—H12B	109.5
C4—C3—H3	109.3	C11—C12—H12C	109.5
C2—C3—H3	109.3	H12A—C12—H12C	109.5
C5—C4—C3	106.17 (13)	H12B—C12—H12C	109.5
C5—C4—H4B	110.5	O1—C13—C14	108.95 (14)
C3—C4—H4B	110.5	O1—C13—H13B	109.9
C5—C4—H4A	110.5	C14—C13—H13B	109.9
C3—C4—H4A	110.5	O1—C13—H13A	109.9
H4B—C4—H4A	108.7	C14—C13—H13A	109.9
C1—C5—C4	107.90 (14)	H13B—C13—H13A	108.3
C1—C5—C6	60.96 (10)	C13—C14—H14B	109.5
C4—C5—C6	115.09 (16)	C13—C14—H14C	109.5
C1—C5—H5	119.6	H14B—C14—H14C	109.5
C4—C5—H5	119.6	C13—C14—H14A	109.5
C6—C5—H5	119.6	H14B—C14—H14A	109.5
O2—C1—C5	119.36 (14)	H14C—C14—H14A	109.5
O2—C1—C6	117.08 (14)	O5—C9—O4	122.20 (18)
C5—C1—C6	60.72 (10)	O5—C9—C10	126.33 (18)
O2—C1—C2	117.65 (13)	O4—C9—C10	111.43 (14)
C5—C1—C2	110.21 (14)	C9—C10—H10A	109.5
C6—C1—C2	119.07 (15)	C9—C10—H10B	109.5
C7—C6—C5	121.31 (16)	H10A—C10—H10B	109.5
C7—C6—C1	119.45 (15)	C9—C10—H10C	109.5
C5—C6—C1	58.32 (11)	H10A—C10—H10C	109.5
C7—C6—H6	115.3	H10B—C10—H10C	109.5
C5—C6—H6	115.3		
			
C15—C20—C19—C18	0.3 (3)	C15—C2—C1—C6	173.81 (14)
C20—C19—C18—C17	−0.8 (3)	C3—C2—C1—C6	51.09 (19)
C19—C18—C17—C16	0.8 (3)	C1—C5—C6—C7	107.50 (18)
C18—C17—C16—C15	−0.4 (3)	C4—C5—C6—C7	−155.14 (15)
C19—C20—C15—C16	0.0 (3)	C4—C5—C6—C1	97.36 (16)
C19—C20—C15—C2	−179.41 (17)	O2—C1—C6—C7	−0.5 (2)
C17—C16—C15—C20	0.0 (3)	C5—C1—C6—C7	−110.66 (18)
C17—C16—C15—C2	179.45 (17)	C2—C1—C6—C7	151.20 (15)
C20—C15—C2—C1	−77.7 (2)	O2—C1—C6—C5	110.16 (16)
C16—C15—C2—C1	102.84 (19)	C2—C1—C6—C5	−98.13 (17)
C20—C15—C2—C3	39.3 (2)	C9—O4—C7—C8	94.3 (2)
C16—C15—C2—C3	−140.13 (17)	C9—O4—C7—C6	−90.12 (17)
C13—O1—C3—C4	76.72 (17)	C5—C6—C7—C8	−9.5 (3)
C13—O1—C3—C2	−164.57 (12)	C1—C6—C7—C8	59.3 (2)
C15—C2—C3—O1	137.72 (15)	C5—C6—C7—O4	175.32 (14)
C1—C2—C3—O1	−100.89 (15)	C1—C6—C7—O4	−115.93 (17)
C15—C2—C3—C4	−99.37 (17)	O4—C7—C8—C21	−4.1 (3)
C1—C2—C3—C4	22.01 (18)	C6—C7—C8—C21	−178.87 (15)
O1—C3—C4—C5	99.09 (17)	C7—C8—C21—C26	−170.96 (17)
C2—C3—C4—C5	−20.44 (18)	C7—C8—C21—C22	8.6 (3)
C3—C4—C5—C1	10.56 (19)	C26—C21—C22—C23	0.3 (3)
C3—C4—C5—C6	−55.11 (19)	C8—C21—C22—C23	−179.20 (16)
C11—O2—C1—C5	144.83 (15)	C21—C22—C23—C24	−0.8 (3)
C11—O2—C1—C6	74.86 (18)	C22—C23—C24—C25	0.2 (3)
C11—O2—C1—C2	−77.26 (18)	C23—C24—C25—C26	0.9 (3)
C4—C5—C1—O2	144.23 (15)	C24—C25—C26—C21	−1.3 (3)
C6—C5—C1—O2	−106.47 (17)	C22—C21—C26—C25	0.7 (3)
C4—C5—C1—C6	−109.30 (17)	C8—C21—C26—C25	−179.71 (16)
C4—C5—C1—C2	3.5 (2)	C1—O2—C11—O3	4.4 (2)
C6—C5—C1—C2	112.78 (17)	C1—O2—C11—C12	−176.47 (14)
C15—C2—C1—O2	−34.6 (2)	C3—O1—C13—C14	165.68 (14)
C3—C2—C1—O2	−157.36 (14)	C7—O4—C9—O5	2.2 (3)
C15—C2—C1—C5	106.86 (16)	C7—O4—C9—C10	−175.71 (14)
C3—C2—C1—C5	−15.86 (19)		
